# Enhancing yield and oil content in oilseed rape hybrids: Insights from line × tester and SIIG approaches

**DOI:** 10.1002/fsn3.4033

**Published:** 2024-02-16

**Authors:** Parastoo Sadat Hashemi, Abdollah Mohammadi, Bahram Alizadeh, Khodadad Mostafavi, Hassan Amiri Oghan

**Affiliations:** ^1^ Department of Agronomy and Plant Breeding, Karaj Branch Islamic Azad University Karaj Iran; ^2^ Oil Crops Research Department, Seed and Plant Improvement Institute Agricultural Research, Education and Extension Organization (AREEO) Karaj Iran

**Keywords:** *Brassica napus*, combining ability, gene action, heritability, ideal genotype selection index

## Abstract

**Background:**

The production of high‐oil‐yielding hybrid varieties is a primary objective in oilseed rape (*Brassica napus* L.) breeding programs. Biometric genetic experiments such as line × tester provide valuable insights into the genetic structure of traits associated with high oil yield.

**Methods:**

In this study, 21 winter hybrids of oilseed rape were evaluated, which were generated by crossing three restorers with seven CMS lines. The experiment was conducted using a line × tester experiment based on a completely randomized block design. Phenological, agronomic, yield, and oil yield components were assessed in this study. The ideal genotype selection index (SIIG) methodology was also employed to identify superior hybrids based on all studied traits simultaneously.

**Results:**

Significant differences were observed between the obtained hybrids and the check cultivars. Heritability analysis revealed that phenological traits were primarily controlled by additive effects, while agronomic and qualitative traits were mainly influenced by non‐additive gene effects. Both broad‐sense and narrow‐sense heritability exhibited a wide range, underscoring the importance of genetic variance. Notably, the hybrids T1 × L5, T1 × L6, and T3 × L1 showed significant specific combining ability values of 394.74, 541.73, and 1236.79, respectively, making them the top specific combinations for increasing seed yield. Based on the SIIG index, hybrids T3 × L1, T1 × L5, T1 × L3, and T2 × L3 emerged as high‐oil‐yielding hybrids with desirable agronomic traits.

**Conclusions:**

The identified superior hybrids by line × tester and SIIG approaches hold promise for the development of high‐yielding oilseed rape cultivars with desirable agronomic traits in oilseed rape breeding programs.

## Graphical abstract

- Line 1 and Tester 2 exhibited the highest positive GCA values for seed yield and oil yield, respectively.
- Crosses T1 × L6 and T3 × L1 demonstrated the highest significant positive SCA values for seed yield and oil yield, respectively.
- T1 × L5 (G5), T1 × L6 (G6), and T3 × L1 (G15) were detected as top‐specific combinations for increasing seed yield.
- T3 × L1 (G15), T1 × L5 (G5), T1 × L3 (G3), and T2 × L3 (G10) emerged as high‐oil‐yielding hybrids with desirable agronomic traits based on the SIIG approach.

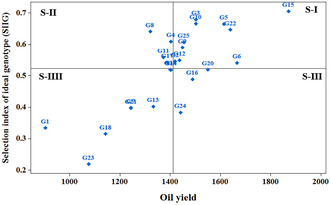

## 1 INTRODUCTION

Oilseed rape (*Brassica napus* L.) is a globally significant oilseed crop, renowned for its economic, industrial, nutritional, social, and ecological advantages (Gül et al., 2007; Masood et al., 2019; Norouzi et al., 2021; Tahir et al., 2018). It ranks as the third‐largest oil plant worldwide, providing edible oil after oil palm and soybean. The wide range of cultivars, including spring, winter, and intermediate types, along with its adaptability to diverse weather conditions, has facilitated its cultivation across the globe (Amiri Oghan et al., 2020; Hegewald et al., 2018). In Iran, oilseed rape production has gained prominence, supported by data from the Food and Agriculture Organization (FAO), which indicates a significant cultivation area dedicated to this crop. According to FAO statistics, in 2021, the cultivation area of oilseed rape in Iran reached **112,729** hectares, reflecting the country's commitment to enhancing its agricultural sector (FAO, 2023).

To sustainably enhance oilseed rape production, particularly in regions like Iran, breeding programs must introduce cultivars with high genetic diversity that are adaptable to different environments (Amiri Oghan et al., 2023). The choice of breeding method depends on trait heritability and gene action type (Rameeh, 2009). Diallele analysis and line × tester analysis are effective methods for evaluating gene functions, general combining ability (GCA), and specific combining ability (SCA), aiding the selection of superior parents in breeding programs (Norouzi et al., 2021; Rashid et al., 2007). These methods have been successfully applied in oilseed rape hybrid production to identify parents with high combining ability and to predict hybrid performance based on their genetic makeup (Ahmad et al., 2022; Kang et al., 2023).

Furthermore, understanding the genetic structure and mode of inheritance for target traits is crucial for improving hybrid or open‐pollinated varieties of oilseed rape. Additive gene effects indicate the efficiency of selection, while non‐additive effects, such as dominance and overdominance, contribute to hybrid variety development (Amiri Oghan et al., 2018; Łopatyńska et al., 2021; Masood et al., 2019). Previous studies have reported high values of broad‐sense heritability, indicating the prevalence of genetic variance over environmental variance for traits such as stem diameter, end of flowering, number of branches, and seed yield (Bidgoli et al., 2021).

Hybrid cultivars play a pivotal role in oilseed rape improvement, offering significant advantages such as increased yield through heterosis, uniform growth, and reduced yield losses due to shattering (Ahmad et al., 2022; Kang et al., 2023). The production of winter oilseed rape hybrids was first achieved using the Ogura method in France by Becker et al. (1998). It has been reported that winter cultivars exhibit 30–40% heterosis for yield, while spring cultivars demonstrate 20–30% heterosis (Becker et al., 1998). Line × tester analysis has been employed to study heterosis and to identify superior hybrid combinations in oilseed rape breeding programs (Rameeh, 2016).

In addition to heterosis, the selection of ideal genotypes is crucial for maximizing yield and agronomic performance in oilseed rape hybrids. The Ideal Genotype Selection Index (SIIG) is a useful tool that combines multiple traits into a single index, enabling breeders to select superior genotypes with desirable characteristics. The SIIG approach considers the relative importance of each trait and assigns appropriate weights to optimize the overall performance of a genotype (Zali et al., 2023). The application of the SIIG method has been successfully demonstrated in oilseed rape breeding to identify genotypes with improved agronomic traits, such as high yield, oil content, and resistance to biotic and abiotic stresses (Abdollahi Hesar et al., 2020; Zali et al., 2015, 2016).

Despite the importance of oil yield and its related traits in oilseed rape breeding, few studies have explored the genetic basis of these traits using biometric genetic methods such as line × tester experiments. Moreover, most of the previous studies have focused on seed yield as the main breeding objective, neglecting oil yield and other agronomic traits that are also relevant for crop productivity and quality. Therefore, there is a need for comprehensive and integrated evaluation of oilseed rape hybrids using both line × tester and ideal genotype selection index (SIIG) approaches to identify superior hybrids with high oil yield and desirable agronomic traits. This research aims to investigate gene action, combinability, heterosis, and the application of the Ideal Genotype Selection Index (SIIG) for traits in oilseed rape hybrids, with the ultimate goal of selecting the best hybrid combinations for improved yield and agronomic performance.

## 2 MATERIALS AND METHODS

### 2.1 Plant materials and experimental methods

This experiment was conducted at the Karaj research farm of the Seed and Plant Improvement Institute of Iran (SPII), located at a latitude of 35 degrees and 48 minutes and a longitude of 51 degrees. The elevation of the farm is 1231 meters above sea level. The study evaluated a total of 21 hybrids of winter oilseed rape, derived from crosses between restorer lines (Testers) and cytoplasmic male sterile (CMS) lines (Lines). The main objective of this study was to investigate the combining ability and heterosis of hybrid combinations. Based on this, 7 CMS lines as lines and 3 restorer lines as testers were crossed. The restorer lines provided after 5 generations of selection from the F2 population were obtained from the Ogura male sterility system and had the ability to restore the fertility of male sterile lines. The restorer lines as testers included three characteristics: (1) 100% purity and dominance (RR), (2) 100% fertility (high ability to produce pollen grains), and (3) having a wide genetic base for restoring fertility. CMS lines were used as female parents and included two characteristics: (1) 100% purity and recessive (rr) and (2) lack of pollen grains. Accordingly, both parental lines were low in their yield potential but pure. The expectation from these crosses was to produce 21 hybrids with high heterosis and 100% fertility. Four check cultivars (Akapi, Ahmadi, Nima, and Nafis) were also included as control varieties (Table 1). The experiment followed a completely randomized block design (RCBD) with three replications conducted over two cropping seasons from 2018 to 2020 (Table 2). Each cropping season is considered as an environment in this study.

**TABLE 1:** Number, name, and hybrid combinations of the studied oilseed rape hybrids.

No.	Hybrid name	Hybrid combination/variety	No.	Hybrid name	Hybrid combination/variety
G1	T1 × L1	WRL‐97‐1*WR‐97‐A1	G14	T2 × L7	WRL‐97‐2*WR‐97‐A7
G2	T1 × L2	WRL‐97‐1*WR‐97‐A2	G15	T3 × L1	WRL‐97‐3*WR‐97‐A1
G3	T1 × L3	WRL‐97‐1*WR‐97‐A3	G16	T3 × L2	WRL‐97‐3*WR‐97‐A2
G4	T1 × L4	WRL‐97‐1*WR‐97‐A4	G17	T3 × L3	WRL‐97‐3*WR‐97‐A3
G5	T1 × L5	WRL‐97‐1*WR‐97‐A5	G18	T3 × L4	WRL‐97‐3*WR‐97‐A4
G6	T1 × L6	WRL‐97‐1*WR‐97‐A6	G19	T3 × L5	WRL‐97‐3*WR‐97‐A5
G7	T1 × L7	WRL‐97‐1*WR‐97‐A7	G20	T3 × L6	WRL‐97‐3*WR‐97‐A6
G8	T2 × L1	WRL‐97‐2*WR‐97‐A1	G21	T3 × L7	WRL‐97‐3*WR‐97‐A7
G9	T2 × L2	WRL‐97‐2*WR‐97‐A2	G22	Okapi	Okapi
G10	T2 × L3	WRL‐97‐2*WR‐97‐A3	G23	Ahmadi	Ahmadi
G11	T2 × L4	WRL‐97‐2*WR‐97‐A4	G24	Nima	Nima
G12	T2 × L5	WRL‐97‐2*WR‐97‐A5	G25	Nafis	Nafis
G13	T2 × L6	WRL‐97‐2*WR‐97‐A6			

**TABLE 2:** Climate and soil data of the eight tested environments.

Cropping seasons	2018–2019		2019–2020	
Accumulated rainfall (mm)	251		369.2	
Average temperature (°C)	15.9		15.2	
Soil Sampling depth (cm)	0–30	30–60	0–30	30–60
Soil electrical conductivity (dS/m)	1.39	1.19	1.33	1.15
Soil pH	7.30	7.10	7.80	7.40
Soil organic carbon (%)	0.87	0.97	0.83	0.96
Soil total nitrogen (%)	0.09	0.04	0.08	0.06
Soil absorbable phosphorus (mg/kg)	14.70	15.60	14.20	15.30
Soil absorbable potassium (mg/kg)	171.00	139.00	165.00	148.00
Soil clay (%)	31.00	26.00	29.00	27.00
Soil silt (%)	44.00	45.00	45.00	46.00
Soil sand (%)	25.00	29.00	26.00	27.00
Soil texture	Clay loam	Clay loam	Clay loam	Clay loam

Before sowing, the seedbed was prepared based on soil testing and fertilizer recommendations. Phosphorus and potassium requirements were met using triple superphosphate (100 kg ha^−1^) and potassium sulfate (200 kg ha^−1^) fertilizers, respectively. Urea was applied at three stages: 2–4 leaves, stem growth, and the beginning of flowering, at rates of 100, 150, and 100 kg ha^−1^, respectively. Systemic pesticides Dimacaron (500 cc ha^−1^) and Ekatin (1000 cc ha^−1^) were used for aphid control, while weed management was done manually. Furrow irrigation was employed in six stages to irrigate the plants.

Each experimental unit consisted of four planting rows, each measuring five meters in length, with a spacing of 30 cm between rows. Agronomic, morphological, phenological, and quantitative traits were measured for each genotype in each replication. Ten random plants were selected for trait measurements. Post‐harvest traits, including yield, were determined by selecting a central portion of the lines after removing 15 cm from the beginning and end. Seed oil content was determined using NMR devices, with 20 grams of seeds collected from each plot. Seed oil yield was calculated by multiplying seed yield (kg ha^−1^) by seed oil content (%), and the result was expressed in kilograms per hectare.

### 2.2 Statistical analysis

The normality of the error distribution was tested using the Kolmogorov–Smirnov test. Line × tester analysis, following the model proposed by Elitriby et al. (1981) and Kempthorne (1957), was used to analyze the data. This analysis provided information on combined variance, general combining ability (GCA) of parents, specific combining ability (SCA) of parents, estimation of gene functions, and determination of trait heritability.

Genetic variance components were calculated using the correlation coefficient. Additive (VA) and dominance (VD) variances were estimated based on the constancy of lines and environments using the following formulas (Singh & Chaudhary, 2007):

$$V_{A}=\left[\left(\text{MSpooled}-\mathrm{MSe}\right)/\mathrm{re}\right]\times \left[4/\left(1+F\right)\right]$$

$$\begin{array}{c} V_{D}=\left[\left({\mathrm{MS}}_{L}\times T-\mathrm{MSe}\right)/\mathrm{re}\right]\times {\left[2/\left(1+F\right)\right]}^{2}\\ \;\left(\text{for composite variance analysis}\right) \end{array}$$

where re is the number of replications, MSe is the mean square of the experimental error, MSpooled is the pooled mean square, and F is the inbreeding coefficient. The pooled mean square (MSpooled) was calculated as follows:

$$\text{MSpooled}=\left({\mathrm{SS}}_{L}+{\mathrm{SS}}_{T}\right)/\left({\mathrm{DF}}_{L}+{\mathrm{DF}}_{T}\right)$$

The mode of gene action was determined by the ratio of the average square of GCA to the square of SCA. The mean degree of dominance was calculated as the square root of the duplicated dominance variance divided by the additive variance. The broad‐sense heritability (h^2^B) and narrow‐sense heritability (h^2^N) of the traits were calculated using the following formulas:

$$h^{2}B=\left(V_{A}+V_{D}\right)/\left(V_{A}+V_{D}+{\mathrm{MS}}_{e}\right)\times 100$$

$$h^{2}N=\mathrm{VA}/\left(V_{A}+V_{D}+{\mathrm{MS}}_{e}\right)\times 100$$

where DF_L_ and DF_T_ are the degrees of freedom for lines and testers, respectively, and MSe is the mean square of the experimental error divided by the number of replications.

The significance of the calculated quantities was determined using t‐tests, comparing the obtained t‐values with the critical t‐value at the given probability level and degrees of freedom of the experimental error (Sendecor & Cochran, 1980). Standard parent heterosis was also calculated, and the significance was tested using the LSD method.

To identify superior genotypes based on all studied traits, the SIIG index (Zali et al., 2023) was utilized, as described below:

$$I=1,2,\ldots ,m\;\text{SIIG}=d_{i}^{-}/d_{i}^{+}+d_{i}^{-}$$

where $d_{i}^{+}$ represents the distance from the ideal genotype and $d_{i}^{-}$ represents the distance from the weak genotype. The SIIG value ranges from zero to one, with a higher value indicating a closer genotype to the ideal genotype. The ideal genotype represents a hypothetical genotype that exhibits optimal performance across all studied traits, while the weak genotype represents a hypothetical genotype that performs poorly across the traits (Zali et al., 2023).

## 3 RESULTS

### 3.1 Combined analysis of variance

A combined analysis of variance was performed to evaluate the effects of different treatments on phenological, agronomic, and qualitative traits. Prior to the analysis, the homogeneity of the variance of errors within each treatment was confirmed using the Fmax statistic (Table 3). The study employed a randomized complete block design (RCBD) for the investigated traits.

**TABLE 3:** The mean square of traits in oilseed rape winter genotypes by line × tester combining analysis method.

S.O.V.	D.f.	MS											
		Initial of flowering	End of flowering	Flowering period	Days to maturity	Plant height (cm)	Number of branches	Number of pods per plant	Number of seeds per pods	1000 seeds weight (g)	Seed yield (kg/ha)	Oil content (%)	Oil seed yield (kg/ha)
Environment (year)	1	717.23**	893.04**	9.63^ns^	211.23**	26,180.90**	0.05^ns^	296,681.61**	288.87**	1.18**	40,447,411.57**	644.81**	10,862,440.26**
Repetition/environment (Error1)	4	13.65	28.81	42.09	12.83	202.55	2.80	1436.80	12.16	0.08	362,874.68	15.09	101,570.02
Genotype	24	21.04**	18.22**	28.36**	11.99**	791.34**	3.31**	19,012.63**	20.26**	0.33**	1,673,134.07**	2.90*	232,753.16**
Check	3	7.33^ns^	7.49^ns^	6.71^ns^	1.26^ns^	53.65^ns^	1.26^ns^	47,361.11**	31.14**	0.23^ns^	2,341,277.49**	0.20^ns^	333,899.13**
Check vs hybrids	1	0.10^ns^	0.08^ns^	0.00^ns^	1.09^ns^	12,797.97**	5.34^ns^	15,613.83^ns^	23.45^ns^	1.50**	171,238.29^ns^	10.20*	1742.22^ns^
Hybrids	20	24.14**	20.74**	33.03**	14.14**	301.66**	3.52**	14,930.30**	18.46**	0.28**	1,648,007.35**	2.94*	229,131.81**
Line	6	33.37**	23.53*	64.22**	14.35**	288.06*	5.02**	24,628.04**	26.71**	0.28*	935,371.39**	2.87^ns^	141,074.01**
Tester	2	54.95**	32.06*	20.29^ns^	21.93**	555.87**	0.01^ns^	32,396.93**	52.05**	0.18^ns^	376,383.41^ns^	0.24^ns^	27,333.21^ns^
Line × tester	12	14.40**	17.46*	19.55*	12.73**	266.09**	3.36*	7170.32^ns^	8.74^ns^	0.30**	2,216,262.65**	3.43*	306,793.81**
Hybrid × environment	20	4.96^ns^	19.19**	30.90**	10.99**	291.59**	3.46**	21,890.39**	8.53^ns^	0.18^ns^	1,136,600.52**	5.36**	176,797.49**
Line × environment	6	4.81^ns^	44.55**	60.59**	3.16^ns^	506.24**	5.71**	33,938.74**	12.00^ns^	0.23^ns^	1,107,655.34**	8.84**	186,813.25**
Tester × environment	2	11.08^ns^	4.39^ns^	27.31^ns^	8.15*	221.95^ns^	4.11^ns^	6251.20^ns^	2.99^ns^	0.01^ns^	1,909,664.66**	8.52**	373,969.15**
Line × tester × environment	12	4.01^ns^	8.98^ns^	16.66^ns^	15.38**	195.88^ns^	2.23^ns^	18,472.75**	7.71^ns^	0.19^ns^	1,022,229.08**	3.09^ns^	138,927.67**
Total error	84	3.70	8.17	10.02	2.28	106.45	1.58	5028.13	7.33	0.11	231,534.73	1.71	30,753.38
Coefficient of variation		1.05	1.31	8.91	0.59	6.20	14.83	25.40	10.87	8.29	12.64	3.55	12.42
Line contribution (%)		41.46	34.03	58.33	30.45	28.65	42.74	49.49	43.39	29.18	17.03	29.27	18.47
Tester contribution (%)		22.76	15.45	6.14	15.51	18.43	0.03	21.70	28.19	6.43	2.28	0.81	1.19
Line × tester contribution (%)		35.78	50.52	35.52	54.04	52.93	57.23	28.82	28.42	64.39	80.69	69.2	80.34

Abbreviation: D.f, degree of freedom; S.O.V, sources of variations.

The results revealed significant impacts of the environment (years) on most of the studied traits, with the exception of the length of the flowering period and the number of branches. Noteworthy differences were observed among the check cultivars in terms of the number of pods per plant, number of seeds per pod, seed yield, and oil yield. Heterosis, determined by comparing the mean square of the check cultivars with that of the hybrids, indicated significant differences in plant height, 1000 seed weight, and seed oil content, highlighting the superiority of the hybrids over the check cultivars.

Significant variations were also observed among the hybrids for the studied traits. The analysis of the hybrids based on line × tester interaction revealed significant differences in the effects of lines, testers, and line × tester combinations for various traits. Furthermore, the interaction effects of hybrid × environment, line × environment, and tester × environment were statistically significant for certain traits, indicating diverse reactions of the genotypes under different environments. Notably, a three‐way interaction effect of line × tester × environment was observed exclusively for the traits of days to maturity, number of branches per plant, seed yield, and mean oil yield, suggesting the presence of gene dominance effects. Additionally, the acceptable coefficient of variation for traits (ranging from 0.59% for days to maturity to 25.40% for the number of pods per plant) indicated the accuracy of the experiment. In this study, line × tester interactions had a higher contribution for most traits than lines and testers. The highest contribution of line × tester was obtained for grain yield and oil yield (80.69% and 80.34%, respectively). This could be due to heterosis in different traits (Table 3).

### 3.2 Mean comparison of genotypes

The mean values of the analyzed traits were compared using the LSD method at 5% and 1% probability levels (Table 4). Several superior hybrids were identified based on their outstanding performance. For early flowering, hybrids T1 × L1 and T1 × L7 demonstrated mean values of 42.50 and 37.33 days, respectively. Similarly, for the longest flowering period, hybrids T1 × L1 and T2 × L1 exhibited mean values of 42.50 and 37.33 days, respectively. Hybrids T2 × L1, T1 × L2, and T3 × L7 displayed the highest number of branches, with mean values of 10.10, 9.60, and 9.60, respectively. Hybrid T2 × L1 exhibited the highest number of pods per plant (391.97), while hybrid T1 × L5 had the highest number of seeds per pod (28.32). Hybrid T3 × L2 showed the highest weight of 1000 seeds (4.28 g). In terms of yield, hybrids T3 × L1, T1 × L6, T3 × L1, and T1 × L6 had the highest values of 5068.40, 4519.79, and 4260.07 kg/ha, respectively. Finally, hybrids T3 × L1 and T1 × L6 demonstrated the highest oil yield, with values of 1870.42 and 1665.93 kg/ha, respectively.

**TABLE 4:** Comparison of the mean traits in oilseed rape winter genotypes by the line × tester method.

Genotypes	Initial of flowering	End of flowering	Flowering period	Days to maturity	Plant height (cm)	Number of branches	Number of pods per plant	Number of seeds per pods	1000 seeds weight (g)	Seed yield (kg/ha)	Oil content (%)	Oil seed yield (kg/ha)
T1 × L1	177.67	220.17	42.50	256.50	157.10	8.77	287.00	24.76	3.86	2447.22	36.46	902.63
T1 × L2	182.67	218.17	35.50	253.50	173.48	9.60	273.50	23.91	3.77	3832.29	36.58	1419.09
T1 × L3	182.67	218.33	35.67	253.83	168.82	8.67	362.43	24.18	4.02	3949.31	37.92	1501.72
T1 × L4	182.33	215.33	33.00	254.83	174.92	8.73	333.07	24.00	3.63	3794.79	36.73	1401.53
T1 × L5	185.00	220.00	35.00	255.33	168.35	8.43	292.00	28.32	4.29	4260.07	37.39	1613.41
T1 × L6	182.67	218.17	35.50	253.17	171.82	6.90	221.00	26.50	3.84	4519.79	36.37	1665.93
T1 × L7	178.67	215.83	37.17	255.00	178.72	8.33	235.77	25.76	3.89	3297.22	36.54	1242.68
T2 × L1	182.00	219.33	37.33	256.67	175.28	10.10	391.97	24.88	3.79	3661.81	35.24	1319.77
T2 × L2	181.33	217.17	35.83	258.00	164.63	7.53	305.60	26.14	4.20	4008.33	36.09	1447.69
T2 × L3	183.00	216.50	33.50	256.17	172.93	9.57	317.27	26.32	3.75	4104.17	36.18	1501.99
T2 × L4	184.33	216.17	31.83	254.00	154.17	8.00	326.00	23.14	3.92	3603.13	37.87	1372.74
T2 × L5	181.67	217.33	35.67	254.67	156.90	7.90	286.50	27.01	4.27	3757.64	37.44	1437.10
T2 × L6	182.67	219.33	36.67	255.67	161.30	7.63	234.20	25.74	3.72	3515.97	37.36	1331.97
T2 × L7	180.33	215.17	34.83	255.50	172.12	8.50	256.80	27.04	3.96	3782.64	36.97	1403.21
T3 × L1	184.00	219.50	35.50	256.50	166.73	8.27	295.97	22.03	4.11	5068.40	36.80	1870.42
T3 × L2	181.67	216.83	35.17	254.67	168.13	8.23	222.60	23.43	4.28	3992.71	37.45	1487.78
T3 × L3	186.00	221.33	35.33	252.33	164.40	8.37	293.60	24.88	3.70	3829.51	36.34	1388.90
T3 × L4	184.33	217.33	33.00	255.00	161.67	8.43	200.53	23.45	3.89	3080.21	37.07	1143.09
T3 × L5	185.00	217.17	32.17	251.67	158.27	8.53	262.63	26.42	3.79	3808.33	36.66	1400.30
T3 × L6	183.33	219.50	36.17	256.50	157.17	7.83	241.57	23.94	4.17	4128.47	37.21	1549.52
T3 × L7	182.67	221.50	38.83	255.00	167.58	9.60	222.60	21.42	4.27	3470.49	35.50	1244.45
Okapi	184.00	218.33	34.33	255.50	139.72	7.90	310.07	28.11	3.64	4336.46	37.41	1638.66
Ahmadi	182.33	219.17	36.83	254.67	137.77	8.33	193.60	23.22	3.44	2845.83	37.66	1075.40
Nima	181.33	216.50	35.17	255.00	143.13	7.33	158.93	27.58	3.89	3829.17	37.26	1441.44
Nafis	182.33	218.17	35.83	255.67	144.22	8.27	342.77	25.07	3.77	3841.32	37.59	1454.16
LSD 5%	2.71	4.02	4.45	2.12	14.51	1.77	99.71	3.81	0.46	676.62	1.84	246.59
LSD 1%	3.59	5.33	5.90	2.81	19.23	2.34	132.15	5.05	0.61	896.76	2.43	326.83

### 3.3 Selection of superior hybrids using the selection index of the ideal genotype (SIIG)

**FIGURE 1:**
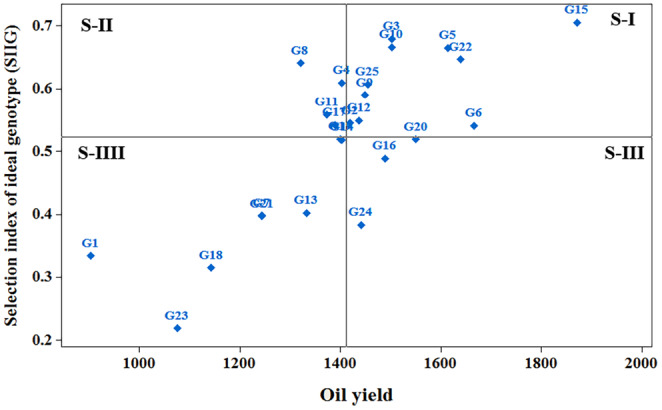
A two‐dimensional distribution diagram of 25 winter oilseed rape genotypes based on oil yield and the SIIG method.

In this research, the ideal genotype selection index (SIIG) was used in order to select the best genotypes using all the investigated traits. The SIIG index was calculated based on the following traits: days to initial flowering, days to end flowering, days to maturity, flowering period length, plant height, number of branches, number of pods per plant, number of seeds per pod, 1000 seed weight, oil percentage, and seed yield. The calculation of the SIIG index assumed that the ideal genotypes had the highest values for plant height, number of branches, number of pods per plant, number of seeds per pod, 1000 seed weight, oil percentage, and seed yield, and the lowest values for days to initial flowering, days to end flowering, flowering period length, and days to maturity. Based on the SIIG index, genotypes G15 (T3 × L1), G3 (T1 × L3), G10 (T2 × L3), G5 (T1 × L5), G22 (Okapi), G8 (T2 × L1), G4 (T1 × L4), G25 (Naïfs), G9 (T2 × L2), and G11 (T2 × L4) were selected as top 10 superior genotypes considering all the studied traits (Table 5). Additionally, to identify genotypes with favorable agronomic traits and oil yield, a two‐dimensional graph of the ideal genotype selection index (SIIG) with oil yield was employed (Figure 1). Based on this analysis, genotypes G15 (T3 × L1), G5 (T1 × L5), G22 (Okapi), G3 (T1 × L3), and G10 (T2 × L3) were selected as superior genotypes, exhibiting higher SIIG values for oil yield. Conversely, genotypes G1 (T1 × L1), G23 (Ahmadi), G18 (T3 × L4), G7 (T1 × L7), G21 (T3 × L7), and G13 (T2 × L6) with lower SIIG values and lower oil yield were identified as weak genotypes in terms of oil yield and other agronomic traits.

**TABLE 5:** Measured distance of genotypes from the desirable genotype (d^+^), unfavorable genotype (d^−^), the ideal genotype selection index (SIIG), and its rank based on all studied traits compared with seed yield and oil yield ranks.

Genotype	Hybrid name	d^+^	d^−^	SIIG index	SIIG rank	Seed yield rank	Oil seed yield rank
G1	T1 × L1	0.221	0.111	0.334	23	25	25
G2	T1 × L2	0.134	0.16	0.545	12	11	13
G3	T1 × L3	0.095	0.202	0.68	2	9	7
G4	T1 × L4	0.116	0.181	0.61	7	15	15
G5	T1 × L5	0.098	0.194	0.665	4	4	4
G6	T1 × L6	0.149	0.175	0.541	14	2	2
G7	T1 × L7	0.178	0.118	0.398	20	22	22
G8	T2 × L1	0.119	0.212	0.641	6	18	20
G9	T2 × L2	0.119	0.171	0.59	9	7	10
G10	T2 × L3	0.097	0.194	0.667	3	6	6
G11	T2 × L4	0.132	0.168	0.559	10	19	18
G12	T2 × L5	0.131	0.159	0.549	11	17	12
G13	T2 × L6	0.173	0.116	0.402	19	20	19
G14	T2 × L7	0.14	0.151	0.518	17	16	14
G15	T3 × L1	0.095	0.228	0.706	1	1	1
G16	T3 × L2	0.154	0.148	0.489	18	8	8
G17	T3 × L3	0.131	0.155	0.542	13	12	17
G18	T3 × L4	0.208	0.096	0.315	24	23	23
G19	T3 × L5	0.14	0.152	0.52	15	14	16
G20	T3 × L6	0.143	0.154	0.519	16	5	5
G21	T3 × L7	0.185	0.122	0.397	21	21	21
G22	Okapi	0.106	0.195	0.647	5	3	3
G23	Ahmadi	0.232	0.065	0.219	25	24	24
G24	Nima	0.203	0.128	0.383	22	13	11
G25	Nafis	0.117	0.18	0.607	8	10	9

### 3.4 General combining ability of lines and testers

The general combining ability (GCA) of lines and testers was estimated for phenological traits, as presented in Table 6. Lines 1 (−1.35**) and 7 (−2.02**) and Tester 1 (−0.90*) exhibited the highest significant negative GCA values for reducing days to initial flowering. Line 4 (−1.83**) displayed the highest negative GCA value for the day to the end of flowering. Only Line 1 (2.91**) showed a significant positive GCA for the length of the flowering period. Lines 3 and 5 exhibited the most significant negative GCA values (−0.87* and − 1.09**, respectively) for reducing days to maturity. Line 5 (−1.02**) represented the most promising general combining ability for the number of branches, while Tester 2 (−0.92*) showed the highest negative GCA value for this trait.

**TABLE 6:** Estimation of the general combining ability effects of lines and testers in terms of traits measured in winter oilseed rape genotypes by the line × tester method.

Genotypes	Initial of flowering	End of flowering	Flowering period	Days to maturity	Plant height	Number of branches	Number of pods per plant	Number of seeds per pods	1000 seeds weight	Seed yield	Oil content	Oil seed yield
L1	−1.35**	1.56*	2.91**	1.58**	−0.03^ns^	0.57^ns^	45.81**	−1.03^ns^	−0.04^ns^	−79.55^ns^	−0.60^ns^	−47.44^ns^
L2	−0.68^ns^	−0.71^ns^	−0.03^ns^	0.41^ns^	2.35^ns^	−0.02^ns^	−11.94^ns^	−0.42^ns^	0.12^ns^	139.09^ns^	−0.06^ns^	39.81^ns^
L3	1.32**	0.62^ns^	−0.70^ns^	−0.87*	2.31^ns^	0.39^ns^	45.26**	0.21^ns^	−0.14^ns^	155.64^ns^	0.04^ns^	52.49^ns^
L4	1.10*	−1.83**	−2.92**	−0.37^ns^	−2.82^ns^	−0.08^ns^	7.36^ns^	−1.39*	−0.15^ns^	−312.65**	0.45^ns^	−105.92*
L5	1.32**	0.06^ns^	−1.25^ns^	−1.09**	−5.23*	−0.18^ns^	1.21^ns^	2.33**	0.16*	136.66^ns^	0.39^ns^	71.89^ns^
L6	0.32^ns^	0.90^ns^	0.58^ns^	0.13^ns^	−2.98^ns^	−1.02**	−46.92**	0.47^ns^	−0.05^ns^	249.39*	0.21^ns^	104.10*
L7	−2.02**	−0.60^ns^	1.41^ns^	0.19^ns^	6.40*	0.34^ns^	−40.78*	−0.18^ns^	0.08^ns^	−288.57*	−0.43^ns^	−114.93**
SE (g_i_)	0.45	0.67	0.75	0.36	2.43	0.30	16.71	0.64	0.08	113.42	0.31	41.33
SE (g_i_‐g_j_)	0.64	0.95	1.06	0.50	3.44	0.42	23.64	0.90	0.11	160.39	0.44	58.46
T1	−0.90**	−0.10^ns^	0.80^ns^	−0.38^ns^	4.05*	0.02^ns^	7.22^ns^	0.43^ns^	−0.06^ns^	−76.69^ns^	0.09^ns^	−19.28^ns^
T2	−0.38^ns^	−0.82^ns^	−0.44^ns^	0.83**	−1.07^ns^	−0.01^ns^	23.45*	0.83*	−0.01^ns^	−29.12^ns^	−0.03^ns^	−9.64^ns^
T3	1.29**	0.92*	−0.37^ns^	−0.45^ns^	−2.98^ns^	−0.01^ns^	−30.67**	−1.26**	0.07^ns^	105.80^ns^	−0.05^ns^	28.93^ns^
SE (g_i_‐g_j_)	0.30	0.44	0.49	0.23	1.59	0.19	10.94	0.42	0.05	74.25	0.20	27.06
SE (g_i_‐g_j_)	0.42	0.62	0.69	0.33	2.25	0.27	15.47	0.59	0.07	105.00	0.28	38.27

For yield‐related traits, Line 1 (2.00**) and Tester 1 (1.61**) demonstrated significant positive GCA values for the number of pods per plant. Line 5 (−1.26**) exhibited the highest negative GCA value for the number of seeds per pod. Line 3 (1.75**) and Tester 2 (1.27*) displayed significant positive GCA values for 1000 seed weight. Line 1 (0.83*) showed the highest positive GCA for seed yield, while Line 6 (−0.82*) indicated the highest negative GCA value for this trait. Tester 1 (1.39**) and Tester 2 (1.26*) exhibited significant positive GCA values for oil yield.

### 3.5 Specific combining ability of line × tester crosses

The specific combining ability (SCA) of line × tester crosses was evaluated to identify the best combinations for the studied traits (Table 7). Cross T3 × L1 displayed significant positive SCA values for days to initial flowering, days to the end of flowering, and length of the flowering period. Crosses T1 × L3 and T2 × L7 exhibited significant negative SCA values for days to maturity. Cross T2 × L1 showed a significant positive SCA value for the number of branches, while cross T1 × L4 had a significant negative SCA value for the same trait.

**TABLE 7:** Estimation of the specific combining ability effects of hybrids in terms of traits measured in winter oilseed rape genotypes by the line × tester method.

Hybrids	Initial of flowering	End of flowering	Flowering period	Days to maturity	Plant height (cm)	Number of branches	Number of pods per plant	Number of seeds per pods	1000 seeds weight	Seed yield	Oil content	Oil seed yield
T1 × L1	−2.65**	0.60^ns^	3.25*	0.33^ns^	−13.33**	−0.30^ns^	−45.20^ns^	0.44^ns^	0.00^ns^	−1201.90**	0.21^ns^	−442.36**
T1 × L2	1.68*	0.88^ns^	−0.80^ns^	−1.51*	0.68^ns^	1.13*	−0.96^ns^	−1.01^ns^	−0.26^ns^	−35.47^ns^	−0.22^ns^	−13.14^ns^
T1 × L3	−0.32^ns^	−0.29^ns^	0.03^ns^	0.10^ns^	−3.95^ns^	−0.22^ns^	30.78^ns^	−1.37^ns^	0.26^ns^	65.00^ns^	1.02^ns^	56.80^ns^
T1 × L4	−0.43^ns^	−0.84^ns^	−0.41^ns^	0.60^ns^	7.28^ns^	0.33^ns^	39.31^ns^	0.04^ns^	−0.12^ns^	378.77^ns^	−0.58^ns^	115.02^ns^
T1 × L5	2.02*	1.49^ns^	−0.08^ns^	1.83**	3.12^ns^	0.13^ns^	4.40^ns^	0.64^ns^	0.23^ns^	394.74*	0.14^ns^	149.09*
T1 × L6	0.68^ns^	−0.73^ns^	−1.41^ns^	−1.56*	4.34^ns^	−0.57^ns^	−18.48^ns^	0.68^ns^	−0.01^ns^	541.73**	−0.70^ns^	169.40*
T1 × L7	−0.98^ns^	−1.56^ns^	−0.58^ns^	0.21^ns^	1.86^ns^	−0.50^ns^	−9.85^ns^	0.59^ns^	−0.09^ns^	−142.87^ns^	0.11^ns^	−34.81^ns^
T2 × L1	1.16^ns^	0.48^ns^	−0.67^ns^	−0.72^ns^	9.98*	1.07*	43.54^ns^	0.15^ns^	−0.12^ns^	−34.89^ns^	−0.90^ns^	−34.86^ns^
T2 × L2	−0.17^ns^	0.60^ns^	0.77^ns^	1.78**	−3.05^ns^	−0.91^ns^	14.92^ns^	0.82^ns^	0.13^ns^	93.01^ns^	−0.58^ns^	5.81^ns^
T2 × L3	−0.51^ns^	−1.40^ns^	−0.90^ns^	1.22^ns^	5.29^ns^	0.71^ns^	−30.61^ns^	0.35^ns^	−0.06^ns^	172.29^ns^	−0.60^ns^	47.43^ns^
T2 × L4	1.05^ns^	0.71^ns^	−0.34^ns^	−1.44*	−8.35^ns^	−0.38^ns^	16.02^ns^	−1.22^ns^	0.12^ns^	139.53^ns^	0.68^ns^	76.60^ns^
T2 × L5	−1.84*	−0.02^ns^	1.83^ns^	−0.06^ns^	−3.20^ns^	−0.38^ns^	−17.33^ns^	−1.08^ns^	0.17^ns^	−155.26^ns^	0.31^ns^	−36.86^ns^
T2 × L6	0.16^ns^	1.15^ns^	0.99^ns^	−0.28^ns^	−1.06^ns^	0.19^ns^	−21.50^ns^	−0.49^ns^	−0.18^ns^	−509.66*	0.41^ns^	−174.19*
T2 × L7	0.16^ns^	−1.52^ns^	−1.67^ns^	−0.50^ns^	0.38^ns^	−0.30^ns^	−5.04^ns^	1.47^ns^	−0.06^ns^	294.97^ns^	0.67^ns^	116.07^ns^
T3 × L1	1.49^ns^	−1.09^ns^	−2.58*	0.40^ns^	3.34^ns^	−0.77^ns^	1.66^ns^	−0.59^ns^	0.12^ns^	1236.79**	0.69^ns^	477.22**
T3 × L2	−1.51^ns^	−1.48^ns^	0.03^ns^	−0.27^ns^	2.37^ns^	−0.22^ns^	−13.96^ns^	0.20^ns^	0.13^ns^	−57.54^ns^	0.79^ns^	7.33^ns^
T3 × L3	0.83^ns^	1.69^ns^	0.87^ns^	−1.33*	−1.33^ns^	−0.49^ns^	−0.16^ns^	1.02^ns^	−0.20^ns^	−237.29^ns^	−0.43^ns^	−104.23^ns^
T3 × L4	−0.62^ns^	0.13^ns^	0.75^ns^	0.84^ns^	1.07^ns^	0.05^ns^	−55.33^ns^	1.19^ns^	0.00^ns^	−518.30**	−0.10^ns^	−191.62**
T3 × L5	−0.17^ns^	−1.92^ns^	−1.75^ns^	−1.77**	0.08^ns^	0.25^ns^	12.93^ns^	0.43^ns^	−0.40**	−239.48^ns^	−0.45^ns^	−112.23^ns^
T3 × L6	0.84^ns^	−0.42^ns^	0.42^ns^	1.84**	−3.28^ns^	0.38^ns^	39.98^ns^	−0.19^ns^	0.19^ns^	−32.08^ns^	0.28^ns^	4.79^ns^
T3 × L7	0.83^ns^	3.08**	2.25^ns^	0.29^ns^	−2.24^ns^	0.80^ns^	14.88^ns^	−2.06^ns^	0.15^ns^	−152.10^ns^	−0.79^ns^	−81.26^ns^
Sca (g_i_)	0.79	1.17	1.29	0.62	4.21	0.51	28.95	1.11	0.13	196.44	0.53	71.59
Sca (g_i_‐g_j_)	1.11	1.65	1.83	0.87	5.96	0.73	40.94	1.56	0.19	277.81	0.75	101.25

For yield‐related traits, cross T1 × L6 demonstrated significant positive SCA values for the number of pods per plant, number of seeds per pod, and seed yield. Crosses T1 × L5 and T2 × L6 displayed significant negative SCA values for the number of pods per plant and the number of seeds per pod, respectively. Cross T3 × L1 showed a significant positive SCA value for 1000 seed weight. Cross T1 × L6 exhibited a significant positive SCA value for seed yield, while cross T2 × L6 had a significant negative SCA value for the same trait. Cross T3 × L1 demonstrated the highest significant positive SCA value for oil yield, while cross T2 × L7 showed a significant negative SCA value for the same trait.

### 3.6 Estimation of standard heterosis

The estimation of standard heterosis for the studied traits is presented in Table 8. Notable findings include hybrids T1 × L1 and T1 × L7, which exhibited significant negative heterosis for the number of days to initial flowering. However, no hybrids showed significant negative heterosis for the day to the end of flowering. Hybrids T1 × L1 and T3 × L7 displayed significant positive heterosis for the length of the flowering period. Some combinations displayed significant negative standard heterosis for days to maturity, while others showed non‐significant effects.

**TABLE 8:** Estimation of standard heterosis of hybrids in terms of traits measured in oilseed rape winter genotypes.

Hybrids	Initial of flowering	End of flowering	Flowering period	Days to maturity	Plant height (cm)	Number of branches	Number of pods per plant	Number of seeds per pods	1000 seeds weight	Seed yield	Oil content	Oil seed yield
T1 × L1	−3.44**	0.46^ns^	15.38**	0.33^ns^	8.93^ns^	5.20**	−16.27^ns^	−11.94**	−0.77**	−43.57^ns^	−3.19**	−44.92^ns^
T1 × L2	−0.72^ns^	−0.46^ns^	−3.62^ns^	−0.85^ns^	20.29**	15.20**	−20.21^ns^	−14.95**	−3.17**	−11.63^ns^	−2.88**	−13.40^ns^
T1 × L3	−0.72^ns^	−0.38^ns^	−3.17^ns^	−0.72^ns^	17.06**	4.00**	5.74^ns^	−13.98**	3.25**	−8.93^ns^	6.69^ns^	−8.36^ns^
T1 × L4	−0.91^ns^	−1.75^ns^	−10.41**	−0.33^ns^	21.29**	4.80**	−2.83^ns^	−14.64**	−6.79**	−12.49^ns^	−2.47**	−14.47^ns^
T1 × L5	0.54^ns^	0.38^ns^	−4.98**	−0.13^ns^	16.73**	1.20^ns^	−14.81^ns^	0.75^ns^	10.23**	−1.76^ns^	−0.73^ns^	−1.54^ns^
T1 × L6	−0.72^ns^	−0.46^ns^	−3.62^ns^	−0.98^ns^	19.14**	−17.20**	−35.52^ns^	−5.75**	−1.37**	4.23^ns^	−3.43**	1.66^ns^
T1 × L7	−2.90*	−1.52^ns^	0.90^ns^	−0.26^ns^	23.92**	0.00^ns^	−31.22^ns^	−8.37**	−0.09^ns^	−23.97^ns^	−2.99**	−24.16^ns^
T2 × L1	−1.09^ns^	0.08^ns^	1.36^ns^	0.39^ns^	21.54**	21.20**	14.35^ns^	−11.51**	−2.70**	−15.56^ns^	−6.43**	−19.46^ns^
T2 × L2	−1.45^ns^	−0.91^ns^	−2.71^ns^	0.91^ns^	14.16*	−9.60**	−10.84^ns^	−7.01**	7.75**	−7.57^ns^	−4.16**	−11.65^ns^
T2 × L3	−0.54^ns^	−1.22^ns^	−9.05**	0.20^ns^	19.91**	14.80**	−7.44^ns^	−6.39**	−3.64**	−5.36^ns^	−3.93**	−8.34^ns^
T2 × L4	0.18^ns^	−1.37^ns^	−13.57**	−0.65^ns^	6.90^ns^	−4.00**	−4.89^ns^	−17.69**	0.64**	−16.91^ns^	0.54^ns^	−16.23^ns^
T2 × L5	−1.27^ns^	−0.84^ns^	−3.17^ns^	−0.39^ns^	8.79^ns^	−5.20**	−16.42^ns^	−3.94*	9.67**	−13.35^ns^	−0.59^ns^	−12.30^ns^
T2 × L6	−0.72^ns^	0.08^ns^	−0.45^ns^	0.00^ns^	11.85^ns^	−8.40**	−31.67^ns^	−8.45**	−4.54**	−18.92^ns^	−0.81^ns^	−18.72^ns^
T2 × L7	−1.99^ns^	−1.83^ns^	−5.43**	−0.07^ns^	19.35**	2.00**	−25.08^ns^	−3.81*	1.80**	−12.77^ns^	−1.83*	−14.37^ns^
T3 × L1	0.00^ns^	0.15^ns^	−3.62^ns^	0.33^ns^	15.61*	−0.80^ns^	−13.65^ns^	−21.63**	5.65**	16.88^ns^	−2.28**	14.14^ns^
T3 × L2	−1.27^ns^	−1.06^ns^	−4.52*	−0.39^ns^	16.58**	−1.20^ns^	−35.06^ns^	−16.67**	10.02**	−7.93^ns^	−0.57^ns^	−9.21^ns^
T3 × L3	1.09^ns^	0.99^ns^	−4.07*	−1.30^ns^	14.00*	0.40^ns^	−14.34^ns^	−11.49**	−5.09**	−11.69^ns^	−3.52**	−15.24^ns^
T3 × L4	0.18^ns^	−0.84^ns^	−10.41**	−0.26^ns^	12.10*	1.20^ns^	−41.50^ns^	−16.58**	−0.21^ns^	−28.97^ns^	−1.58*	−30.24^ns^
T3 × L5	0.54^ns^	−0.91^ns^	−12.67**	−1.56^ns^	9.74^ns^	2.40**	−23.38^ns^	−6.02**	−2.57**	−12.18^ns^	−2.67**	−14.55^ns^
T3 × L6	−0.36^ns^	0.15^ns^	−1.81^ns^	0.33^ns^	8.98^ns^	−6.00**	−29.52^ns^	−14.86**	7.15**	−4.80^ns^	−1.21^ns^	−5.44^ns^
T3 × L7	−0.72^ns^	1.06^ns^	5.43**	−0.26^ns^	16.20**	15.20**	−35.06^ns^	−23.81**	9.59**	−19.97^ns^	−5.75**	−24.06^ns^
SE (Het)	1.11	1.65	1.83	0.87	5.96	0.73	40.94	1.56	0.19	277.81	0.75	101.25

For the number of branches, hybrids T1 × L2, T2 × L1, T2 × L3, and T3 × L7 exhibited the most significant positive standard heterosis. Directional dominance was observed for most traits, with some hybrids displaying positive but non‐significant heterosis for the number of pods per plant and the number of seeds per pod. No significant positive heterosis was observed for seed yield, seed oil content, and oil yield. However, it is worth noting that 1000 seed weight was identified as an influential trait for oilseed rape seed yield. Hybrids such as T1 × L5, T3 × L7, T3 × L2, and T3 × L6 demonstrated the highest positive standard heterosis for 1000 seed weight.

### 3.7 Heritability and mode of gene action

Table 9 represents the estimates of broad‐sense and narrow‐sense heritability, as well as the mode of gene action for phenological, agronomic, and qualitative traits. Broad‐sense heritability exhibited a wide range, with values ranging from 34.86% for seed oil content to 87.91% for days to initial flowering. The high values of broad‐sense heritability indicate that genetic variance plays a more significant role than environmental variance in determining these traits.

**TABLE 9:** Estimation of variance components, broad‐sense and narrow‐sense heritability (%), and action of gene traits in oilseed rape winter genotypes by the line × tester method.

Variance components and parameters	Initial of flowering	End of flowering	Flowering period	Days to maturity	Plant height (cm)	Number of branches	Number of pods per plant	Number of seeds per pods	1000 seeds weight	Seed yield	Oil content	Oil seed yield
Additive variance	11.69	5.83	14.41	4.66	82.85	0.73	7180.71	8.57	0.05	188,029.89	0.17	27,295.14
Dominance variance	1.78	1.55	1.59	1.74	26.61	0.30	357.03	0.24	0.03	330,787.99	0.29	46,006.74
Broad‐sense heritability (%)	87.91	64.36	76.15	84.88	67.28	56.47	74.99	70.61	60.01	81.76	34.86	82.66
Narrow‐sense heritability (%)	76.28	50.85	68.58	61.76	50.93	40.14	71.44	68.72	35.73	29.63	12.92	30.78
The average degree of dominance of genes	0.55	0.73	0.47	0.87	0.80	0.90	0.32	0.23	1.17	1.88	1.84	1.84
Vargca/Varsa	3.28	1.88	4.53	1.34	1.56	1.23	10.06	18.20	0.74	0.28	0.29	0.30

Narrow‐sense heritability also showed a diverse range. The lowest narrow‐sense heritability was observed for seed yield (36.24%), seed oil content (37.06%), and seed oil yield (37.24%). On the other hand, phenological traits such as days to initial and end of flowering (86.77% and 79.01%, respectively), days to maturity (72.76%), and yield components, including plant height (75.69%), number of pods per plant (95.26%), and number of seeds per pod (97.33%), exhibited the highest narrow‐sense heritability.

The mean degree of dominance for most phenological and agronomic traits was less than one, except for 1000 seed weight, seed yield, oil content, and seed oil yield.

## 4 DISCUSSION

Oilseed rape (*Brassica napus*) breeding programs aim to develop superior genotypes with desirable traits that can thrive in diverse environmental conditions. Understanding the influence of environmental factors on trait performance and the genetic diversity within cultivars is crucial for effective breeding strategies. Furthermore, the evaluation of hybrids in comparison to check cultivars provides insights into their potential for improved agronomic traits and yield. The selection index of ideal genotype (SIIG) in combination with the analysis of lines for their general and specific combining abilities (GCA and SCA) contributes to the identification of superior oilseed rape genotypes. Estimating standard heterosis and assessing heritability and the mode of gene action further enhance our understanding of the genetic basis of trait variation in oilseed rape breeding.

### 4.1 Environmental influence and genotypic variation: Implications for oilseed rape breeding

The results of this study align with previous research highlighting the significant influence of environmental factors on the performance of studied traits in oilseed rape (Perveen et al., 2005; Tuncturk et al., 2005). Environmental effects on traits such as days to flowering, days to maturity, and yield have been reported in recent studies as well (Bakhshi, Amiri Oghan, Rameeh, Fanaei, et al., 2023; Bakhshi, Amiri Oghan, Rameeh, Zeinalzadeh Tabrizi, et al., 2023). This emphasizes the need for multi‐environment trials to assess trait stability and adaptability across different growing conditions. Consistent with current literature, the present study identified significant genotypic variation in all the studied traits, indicating the presence of high genetic diversity among the tested oilseed rape genotypes (Bakhshi, Amiri Oghan, Rameeh, Zeinalzadeh Tabrizi, et al., 2023). Genetic variation serves as the foundation for selecting superior genotypes with desirable traits and is instrumental in breeding programs aimed at improving oilseed rape.

### 4.2 Performance of hybrids and check cultivars: Insights for enhancing oilseed rape productivity

The performance of hybrids in this study aligns with recent findings, where hybrids demonstrated significant variation in various agronomic and yield‐related traits (Wolko et al., 2019). The analysis of line × tester effects confirmed the substantial contributions of both parental lines and testers to the performance of hybrids. This underscores the importance of selecting appropriate parental lines and testers to obtain desirable traits in oilseed rape hybrids. Comparison with check cultivars further supports recent research, showing significant differences between hybrids and check cultivars in traits such as pod number, seed number per pod, seed yield, and oil yield (Szała et al., 2019). The superiority of hybrids over check cultivars in these traits highlights the potential for achieving higher productivity and oil production through hybrid breeding in oilseed rape.

### 4.3 Selection of superior genotypes using the selection index of ideal genotype (SIIG) in oilseed rape breeding

The SIIG index is a selection model that helps the researcher choose the most ideal genotype(s) among the studied genotypes based on the traits of interest (Zali et al., 2023). The SIIG index integrated the studied traits into a single index, which allowed us to select superior genotypes more reliably and accurately. The use of the SIIG as a tool for selecting superior genotypes with desirable combinations of traits is well established in current oilseed rape research (Abdollahi Hesar et al., 2020; Zali et al., 2015, 2016). Recent studies have successfully employed SIIG to integrate multiple traits and facilitate the selection of superior genotypes (Gholizadeh et al., 2021; Zali et al., 2023). The SIIG analysis conducted in this study identified genotypes with higher SIIG values for oil yield and agronomic traits as superior genotypes, including G15 (T3 × L1), G5 (T1 × L5), G22 (Okapi), G3 (T1 × L3), and G10 (T2 × L3).

### 4.4 Unraveling specific combining ability (SCA) in oilseed rape: Optimizing hybrid combinations for trait improvement

Specific combining ability refers to the interaction between two specific parental lines and their performance when combined in a hybrid. In oilseed rape breeding, SCA analysis helps identify hybrid combinations that exhibit significant effects on various traits (Teklewold & Becker, 2005). By assessing SCA, breeders can determine which combinations are most effective in achieving desired trait improvements. The significant SCA observed for traits such as flowering time, plant height, number of branches, grain yield, and oil yield indicates that certain hybrid combinations have a specific advantage in manipulating these traits (Ramee et al., 2009, 2016). For instance, hybrids T1 × L1 and T2 × L5 showed significant SCA for reducing the days to initial flowering, while hybrids T1 × L1 and T2 × L4 were recognized as the best combinations for reducing plant height. These hybrids can be valuable in breeding programs that aim to modify these traits. Additionally, hybrids T1 × L2 and T2 × L1 exhibited the best private combinations for increasing the number of branches. This finding aligns with previous studies that have also reported specific combining ability for branch traits in oilseed rape hybrids (Kang et al., 2023). Identifying hybrids with significant SCA for desired traits allows breeders to select appropriate parental lines for hybridization, leading to more efficient and targeted trait improvement.

### 4.5 Standard heterosis estimation in oilseed rape breeding: Assessing hybrid performance

Standard heterosis estimation provides insights into the performance of hybrids compared to their parents. Heterosis, also known as hybrid vigor, refers to the phenomenon where hybrids exhibit superior performance or traits compared to their parents (Teklewold & Becker, 2005). By estimating standard heterosis, breeders can assess the extent of improvement achieved through hybridization. In oilseed rape breeding, certain hybrids displayed significant positive heterosis for traits such as flowering time and the number of branches (Fang et al., 2022). This indicates that these hybrids outperformed their parents in these specific traits. For example, hybrids T1 × L1 and T1 × L7 showed significant positive heterosis for the length of the flowering period, while hybrids T1 × L2, T2 × L1, T2 × L3, and T3 × L7 exhibited the most significant positive heterosis for the number of branches per plant. However, it is important to note that not all traits showed significant positive heterosis. Traits like seed yield, seed oil content, and oil yield did not exhibit significant positive heterosis in the studied hybrids. This implies that other factors, such as additive genetic effects or selection for different traits like 1000 seed weight, may be more effective in improving oilseed rape yield (Fang et al., 2022; Kang et al., 2023).

### 4.6 Heritability and mode of gene action in oilseed rape: Understanding genetic control of traits

Heritability measures the proportion of phenotypic variation in a trait that can be attributed to genetic factors. In oilseed rape breeding, understanding heritability helps determine the relative importance of genetic and environmental factors in controlling various traits. The estimation of broad‐sense and narrow‐sense heritability provides insights into the contribution of genetic and environmental variance to the studied traits (Amiri Oghan et al., 2018). High broad‐sense heritability values indicate that genetic factors play a more significant role than environmental factors in controlling these traits. For example, the high broad‐sense heritability observed for days until initial flowering suggests that genetic variance primarily influences this trait. On the other hand, narrow‐sense heritability estimates the contribution of non‐additive genetic effects, such as dominance and epistasis. Traits like phenological traits (e.g., days to initial and end of flowering) and yield components (e.g., number of pods per plant, number of seeds per pod) showed high narrow‐sense heritability values. This suggests that non‐additive genetic effects play a crucial role in controlling these traits. Considering traits with high heritability, particularly those with high narrow‐sense heritability, could lead to more efficient selection and breeding programs. By continuously selecting and improving these traits through recurrent selections, oilseed rape lines with higher yield potential can be developed (Amiri Oghan et al., 2018; Kang et al., 2023).

## 5 CONCLUSION

In conclusion, this study on the evaluation of oilseed rape hybrids provides valuable insights into the genetic structure of traits associated with high oil yield, contributing to the advancement of oilseed rape breeding programs. The identified hybrids, particularly T3 × L1, T1 × L5, T1 × L3, and T2 × L3, hold promise for the development of high‐yielding oilseed rape cultivars with desirable agronomic traits. This study highlighted the significant influence of environmental factors on trait performance and the presence of high genetic diversity among the tested genotypes. The evaluation of hybrids compared to check cultivars underscores the potential for achieving higher productivity and oil production through hybrid breeding in oilseed rape. The novelty of this study lies in the application of both line × tester and SIIG methods to identify high‐oil‐yielding hybrids with desirable agronomic traits in oilseed rape. These methods are useful for revealing the genetic structure of traits associated with high oil yield, as well as for selecting superior genotypes and hybrid combinations. This report is the first study that combines these two methods in oilseed rape breeding. This study also provided novel insights into the genetic diversity, heterosis, heritability, and gene action of oilseed rape hybrids, which can facilitate the development of improved breeding strategies and cultivars. These findings contribute to the enhancement of crop productivity in oilseed rape, fostering the development of improved cultivars to meet the growing demands of the oilseed industry. However, future studies should expand the scope and scale of oilseed rape hybrid evaluation, using larger and more diverse genotype collections and considering other important traits such as disease resistance, abiotic stress tolerance, and quality attributes.

## AUTHOR CONTRIBUTIONS

**Parastoo Sadat Hashemi:** Investigation (equal); writing – original draft (equal); writing – review and editing (equal). **Abdollah Mohammadi:** Project administration (equal); supervision (equal); validation (equal). **Bahram Alizadeh:** Project administration (equal); resources (equal); supervision (equal); writing – review and editing (equal). **Khodadad Mostafavi:** Project administration (equal); supervision (equal); validation (equal). **Hassan Amiri Oghan:** Formal analysis (equal); methodology (equal).

## FUNDING INFORMATION

This research received no specific grant from any funding agency in the public, commercial, or not‐for‐profit sectors.

## CONFLICT OF INTEREST STATEMENT

The authors declare that they have no conflict of interest.

## INFORMED CONSENT

Written informed consent was obtained from all study participants.

## Data Availability

The data that support the findings of this study are available from the corresponding author upon reasonable request.
